# Microbial ecology of biofiltration used for producing safe drinking water

**DOI:** 10.1007/s00253-022-12013-x

**Published:** 2022-06-30

**Authors:** Xi Bai, Inez J. T. Dinkla, Gerard Muyzer

**Affiliations:** 1 Microbial Systems Ecology, Department of Freshwater and Marine Ecology, Institute for Biodiversity and Ecosystem Dynamics, University of Amsterdam, 1098 XH Amsterdam, The Netherlands; 2 Wetsus, European Centre of Excellence for Sustainable Water Technology, Oostergoweg 9, 8911 MA Leeuwarden, The Netherlands

**Keywords:** Drinking water treatment, Biofiltration, Microbial ecology, Biofilter removal performance

## Abstract

**Abstract:**

Biofiltration is a water purification technology playing a pivotal role in producing safe drinking water. This technology attracts many interests worldwide due to its advantages, such as no addition of chemicals, a low energy input, and a high removal efficiency of organic compounds, undesirable taste and odours, and pathogens. The current review describes the microbial ecology of three biofiltration processes that are routinely used in drinking water treatment plants, i.e. (i) rapid sand filtration (RSF), (ii) granular activated carbon filtration (GACF), and (iii) slow sand filtration (SSF). We summarised and compared the characteristics, removal performance, and corresponding (newly revealed) mechanisms of the three biofiltration processes. Specifically, the microbial ecology of the different biofilter processes and the role of microbial communities in removing nutrients, organic compounds, and pathogens were reviewed. Finally, we highlight the limitations and challenges in the study of biofiltration in drinking water production, and propose future perspectives for obtaining a comprehensive understanding of the microbial ecology of biofiltration, which is needed to promote and optimise its further application.

**Key points:**

• *Biofilters are composed of complex microbiomes, primarily shaped by water quality*.

• *Conventional biofilters contribute to address safety challenges in drinking water*.

• *Studies may underestimate the active/functional role of microbiomes in biofilters*.

**Supplementary Information:**

The online version contains supplementary material available at 10.1007/s00253-022-12013-x.

## Introduction

Drinking water sources including surface water from rivers, lakes, and reservoirs, as well as groundwater aquifers, may contain a variety of contaminants, such as organic compounds, chemical substances, and pathogenic viruses, bacteria, and protozoa (Pandey et al. 2014; Palansooriya et al. 2020). Due to increasing human activities, the safety of drinking water sources is currently facing enormous challenges worldwide and has been associated with increased risk of contamination (Shannon et al. 2008; World Health Organization 2017; Zhang et al. 2017; Favere et al. 2021). Contamination of drinking water can lead to waterborne diseases (e.g. cholera, typhoid, hepatitis A, and dysentery) and, hence, threatens public health worldwide (Moreira and Bondelind 2017; World Health Organization 2017). To address drinking water-related health problems, processes of establishing sufficient barriers against contaminants in water sources, further referred to as water treatment, is essential prior to consumption.

Biofiltration treatment is now attracting more interest worldwide due to its advantages of avoiding addition of chemicals, low energy input, and higher removal efficiency in turbidity, organic compounds, undesirable tastes and odours, and especially pathogens (e.g. bacteria, viruses, and protozoa), meanwhile meeting the increased demand of safe and high-quality drinking water (Haig et al. 2011; Nyberg et al. 2014; Basu et al. 2015; Zhang et al. 2017; Oh et al. 2018; Maurya et al. 2020). Biofiltration is a process that not only removes fine particles through physicochemical means (e.g. straining and sorption) like other conventional filters but also captures and degrades contaminants through biological activities (Basu et al. 2015; Liu et al. 2017; Terry and Summers 2018). It has been used in Europe for purifying surface water to effectively reduce turbidity and cholera bacteria in drinking water applications since the early 1900s. However, the importance of biofiltration in drinking water treatment was noticed only after it was found to benefit in reducing microbial growth (in the distribution pipelines), corrosion potential, and the disinfection by-products a few decades ago (Chaudhary et al. 2003). With the increasing presence of so-called contaminants of emerging concern (CECs), such as pharmaceutical residues and industrial chemicals in source waters (Zhang et al. 2017; Yusuf et al. 2021), a good understanding of biofiltration processes is essential for further improvement in the production of safe drinking water.

Here, we comprehensively review and compare 3 major biofiltration processes in drinking water treatment plants (DWTPs), including rapid sand filtration (RSF), granular activated carbon filtration (GACF), and slow sand filtration (SSF), from both the performance and mechanistic perspectives. The review specifically focusses on the microbial ecology of biofilters and the role of microbial communities in the removal of nutrients, organic compounds, and pathogens. In addition, the limitations, biases, and challenges of the research to date are discussed. This study proposes directions that will complement current studies to obtain a comprehensive understanding of the microorganisms involved in the production of safe drinking water.

## Biofilters in the drinking water treatment process

**Fig. 1:**
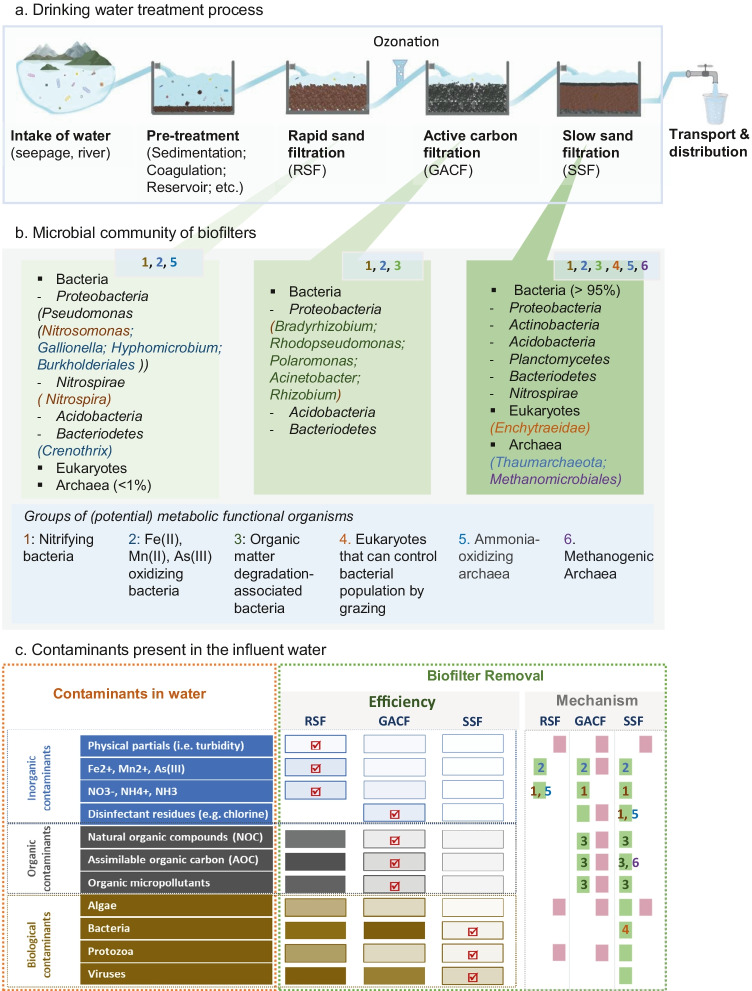
Conceptional model of biofiltration processes, the microorganisms present, and the possible mechanisms in the removal of different contaminants. **a** Overview of the different biofiltration processes in the production of drinking water. **b** Microbial community of biofilters. A summary of microorganisms present in biofilters is listed in 3 green boxes (including dominantly occurring bacterial phyla). Potentially functional organisms are described in the right box. The occurrence of potentially functional groups of organisms is indicated by the numbers below each box. Examples of functional groups that exist in the group are indicated by corresponding colours under the phylum. **c** Contaminants present in the influent water (orange box) grouped into inorganic, organic, and biological contaminants. Biofiltration removal efficiency (green box, left columns): the intensity of the colour represents the relative amount of the contaminant in the effluent water, i.e. the more intense, the more contaminants break through the filters and will be present in the effluent water, and so less removal efficiency; conversely, the lighter the column, less contaminants enter the effluent, and the better removal efficiency;  highlight the major removal of the biofiltration step. Biofilter removal mechanisms (green box, right columns): the mechanisms involved in the removal of the corresponding contaminants are grouped and presented via  Biological removal and/or  Physical–chemical removal. The numbers marked on the colour block (1–6, explained in **b**) represent the organism groups involved in the removal process

Since the early twentieth century, multiple-stage water treatments are carried out in the majority of DWTPs. DWPTs comprise 3 main biofiltration processes including RSF, GACF, and SSF, which are applied together with coagulation, flocculation, sedimentation, and disinfection steps in various combinations (Fig. 1a) (Basu et al. 2015; World Health Organization 2017).

### Filtration performance of biofilters

#### Rapid sand filtration

RSF processes water with a wide range of initial turbidity at a high flow rate (5–30 m/h), which can reduce most physical hazards (particles) and inorganic compounds (e.g. iron, manganese, ammonia, nitrate) (World Health Organization 2017; Brandt et al. 2017). In a combination of pre-treatments including coagulation and sedimentation, an appropriate RSF process can reduce the water turbidity to less than 0.1 nephelometric turbidity units (NTU) (World Health Organization 2017). When the water turbidity is reduced to ≤ 0.3 NTU in 95% of the samples and none to exceed 1 NTU, about 1–2 log_10_ reduction of viruses and 3 log_10_ reductions of *Cryptosporidium* are considered to be achieved (World Health Organization 2017; Brandt et al. 2017)*.* Besides, RSF has a proven effect on removing iron (28.6–97%) and manganese (50–97%) and to a less extent on removing unpleasant odour and taste, bacteria (0.3–3 log_10_), and organic matters (e.g. 99% methane) (Bishaw and Kebede 1999; Lee et al. 2014; Asami et al. 2016; Poghosyan et al. 2020) (Table S1).

#### Granular activated carbon filtration

GACF is applied to further remove chemical and biological hazards that can break through the RSF and the following ozonation barriers. Usually, water is first treated by ozone after RSF and then by GACF at a relatively fast flow rate (6−7.5 m/h) (World Health Organization 2017; Brandt et al. 2017; de Vera et al. 2019).

GACF is primarily designed to remove unpleasant odour, taste, and colour from water caused by natural organic matter (NOM) and organic micropollutants (Magic-Knezev et al. 2008; Simpson 2008). In the last decades, GACF has been observed to effectively remove a wide range of CECs (e.g. 22 to > 80% of 16 studied CECs including pharmaceutical residues, pesticides, and fire retardant) and assimilable organic carbon (AOC) (39−74%) associated with microbial stability of water (Wang et al. 2013; World Health Organization 2017; Zhang et al. 2017; Greenstein et al. 2018; de Vera et al. 2019; Pick et al. 2019) (Table S3). Regarding microbial contaminants, GACF removes protozoan (oo)cyst (1.0−2.3 log_10_) such as *Cryptosporidium parvum* and *Giardia lamblia*. However, GACF shows limited removal efficiency of faecal indicator bacteria (i.e. ≤ 0.5−1.1 log_10_ *Escherichia coli*) and anaerobic spores (0.9−1.1 log_10_ spores of *Clostridium bifermentans*), and even lower removal efficiency of viruses and bacteriophages (0−0.7 log_10_ MS2 phage) (Hijnen et al. 2010; Hijnen and Medema 2010).

#### Slow sand filtration

Differing from RSF filters, finer sand is the filter medium in SSF. The sand bed filters water with a low flow rate (0.1−0.5 m/h) (Brandt et al. 2017). SSF is effective for reducing turbidity, total organic carbon (TOC), nitrogen compounds, pesticides, pharmaceutical chemicals, and microbial contaminants (Table S5) (Hijnen et al. 2004; Bichai et al. 2010; D'Alessio et al. 2015). SSF is one of the oldest and most effective approaches to control microbial contamination (i.e. pathogenic oocysts, bacteria, and viruses) from drinking water (Hijnen et al. 2007; Haig et al. 2011). SSF typically removes 2−6 log_10_ oocysts, 2−4 log_10_ of bacteria, and < 1−3 log_10_ of viruses (Hijnen et al. 2004; Wakelin et al. 2010; Matuzahroh et al. 2020) (Table S5).

SSF performance depends on source water characteristics, temperature, sand type, grain size, bed depth, filtration rate, and the age and thickness of the “Schmutzdecke” (Matuzahroh et al. 2020; Maurya et al. 2020; Schijven et al. 2013; Yogafanny et al. 2014). The removal efficiency increases along with a finer grain size, a longer bed depth, a slower flow rate, and an older age of Schmutzdecke (Bauer et al. 2011; Schijven et al. 2013; Yogafanny et al. 2014). The Schmutzdecke (SD) is a slimy biofilm layer on top of the slow sand filter with a thickness of 0.5 up to 3 cm, which is described in more detail in the later sections.

### Mechanism of biofiltration processes

**Fig. 2:**
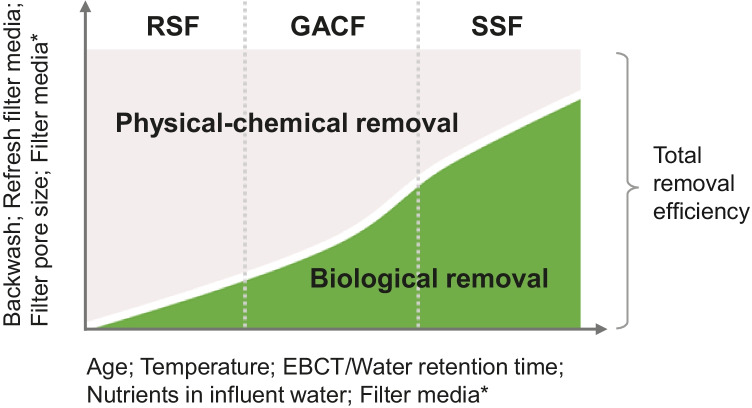
Conceptional model of dynamic accumulation of removal mechanisms in RSF, GACF, and SSF filters. The *x*-axis lists the conditions that could stimulate (positive correlate to) biological removal; The *y*-axis lists conditions that can increase the physical–chemical removal portion. The white curve depicts the dynamic changes in the proportion of physical–chemical and biological removal mechanisms in the total removal performance. The change in slope (in GACF) refers to the transition from normal GACF to biological-active GACF. *Depending on material characteristics, filter media could positively induce both the physical–chemical and biological removal

In biofiltration processes, the biological removal always occurs in parallel with physical–chemical removal (Wu et al. 2014; Verma et al. 2017), although one removal mechanism could outperform the other one (Fig. 2).

When operating a new filter, the removal is initially achieved through the physical mechanisms straining, and adsorption (Hijnen and Medema 2010; Knezev 2015). Meanwhile, suspended particles gradually form a nutrient-rich rough layer on the filter surface, attracting microorganisms to colonise the surface. Biofilms are then generated on the surface over time. From this stage onwards, biological removal (e.g. biodegradation) gradually becomes the main removal mechanism (Hammes et al. 2008; Velten et al. 2011; Brown et al. 2015).

Biological removal depends on the biological activity of the biotic community accumulated in filters. Various operating conditions of the filtration process can affect the biotic community, such as filter media, nutrients from influent water, flow rate, temperature, and maintenance measures (i.e. backwashing and filter media replacement), resulting in unique removal mechanism patterns of RSF, GACF, and SSF (further discussed in “Microbial communities in biofilters and its potential functions in biological removal”) (Fig. 2).

In RSF, large amounts of nutrients are introduced into the filter bed, supporting rapid accumulation of the microbial community. This community includes nitrifying bacteria, methane-oxidising bacteria, iron-oxidising bacteria, and heterotrophic bacteria that were found to play important roles in degrading and removing ammonium, methane, and bentazone and in co-metabolising various organic micropollutants (such as CECs and naturally produced organic compounds) (de Vet et al. 2011; Wang et al. 2020). However, biomass accumulation (and also metal precipitates) in the filter leads to clogging and thus decreases the filtration rate over time (Maurya et al. 2020). To extend the life of RSF filters, backwashing (i.e. pumping water back into the filter media) has to be performed frequently every few hours up to days (flow rate varies from 1 to 20 m/h) (Hammes et al. 2011). As a result, biomass including microbial communities and their produced extracellular substances (EPS), and other residue compounds are promptly discarded from the filter, resulting in temporary loss of filter performance. Usually, RSF can be put back into operation instantly after cleaning (or within 15–60 min) (Brandt et al. 2017; Bruni and Spuhler 2012). This is due to biological removal only contributes to a limited extent in RSF (Fig. 2), and the effect of backwashing on RSF filter performance is usually not considered significant (Prevost et al. 2005). Owing to the introduction of higher nutrient concentrations from less treated influent water in RSF, recovery of the low biological removal contribution to its pre-cleaning state usually takes a shorter time.

In GACF and SSF, the filters are cleaned less frequently, i.e. GACF is backwashed weekly in summer and monthly in winter (Gibert et al. 2013; Knezev 2015); SSF is usually cleaned by scraping the SD layer every few months or even years (Schijven et al. 2013; Chan et al. 2018). Therefore, microbial communities can accumulate in the filters with less disturbance and contribute to biological removal. However, after applying the cleaning process, the recovery of the biological filter performance is, due to the relatively high ratio of biological removal contributions (Fig. 2), slower in GACF and SSF than RSF. Backwashing has been reported to have a significant impact on the community structure of biofilms accumulated on GACF media, causing a decrease in microbial diversity, and about half of the biomass can be washed away during backwashing (Liao et al. 2013a, 2015). Within a few days after backwashing and usually before the next backwash cycle, the biomass (probably related to biodegradation performance) returns to the pre-backwash concentration (Gibert et al. 2013; Liao et al. 2013a; Qi et al. 2019). In SSFs, the new Schmutzdecke layer takes some time to form and ripen after scraping. This usually takes a few days to weeks, and when the processed water can meet the requirement again, the filter can be used again for production (Burch and Thomas 1998; De Souza et al. 2021).

GAC has a high affinity for organic compounds because of the large surface area (500–1500 m^2^/g). GACF could physically filter pollutants through adsorption, attachment, and straining (Hijnen and Medema 2010; Knezev 2015). The flow rate controls the contact time between water and carbon, which is expressed as the Empty Bed Contact Time (EBCT). This parameter is widely used to estimate the removal performance of GACF. Previously, when the adsorption capacity of GAC is exhausted (i.e. expressed as a higher EBCT), the GAC is replaced or regenerated to maintain the desired removal performance of the GACF. With the recognition that increasing EBCT can improve biological removal, biofiltration features have been considered to contribute to the GACF performance (Brown et al. 2015; Oh et al. 2018). DWTPs that do not regularly change their filter media naturally change into biologically active GAC filters. At this stage, the adsorption capacity of the GAC is gradually depleted. At the same time, nutrients are continuously introduced into the filter to support the continued development of the microorganisms, which results in a rapid increase in the proportion of biological removal in the total removal contribution (Fig. 2) (Hammes et al. 2008; Velten et al. 2011; Brown et al. 2015). Regarding the BACF removal of organic carbon, chemical contaminants, and microbial contaminants (e.g. oocysts, bacteria, etc.), absorption removal is usually more efficient than biological removal due to the high-affinity properties of GAC materials. However, the biological removal specifically targets the biodegradable organic carbon fraction such as formaldehyde, geosmin, and compounds that are not identifiable, e.g. biodegradable humid substances (Hijnen and Medema 2010; Velten et al. 2011; Knezev 2015; Terry and Summers 2018). So far, limited predation of pathogens has been studied in GASF (Hijnen et al. 2007).

Differing from RSF and GACF, newly installed SSF filters cannot be used directly. SSF filters must be subjected to several months of ripening. During this ripening period, the so-called “Schmutzdecke”, which is a brown slimy layer or biofilm ranging from 0.5 to 2.0 cm (up to 3 cm) thick is formed on the surface of the filter (Matuzahroh et al. 2020; Ranjan and Prem, 2018). It is reported that sufficient biohazard removal efficiency (i.e. removal of pathogens from the water and reduction of nutrients causing poor biostability) can be achieved by a Schmutzdecke older than 6 months old (mature Schmutzdecke) to ensure that the treated water meets the desired quality requirements (Chaudhary et al. 2003; Bauer et al. 2011; Brandt et al. 2017).

A mature Schmutzdecke layer consists of a diverse community of bacterial, archaeal, and eukaryotic microorganisms, EPS produced by the microorganisms, and other organic and inorganic debris and particulate matter (Pontius 2003; Wakelin et al. 2010). The mature Schmutzdecke layer is very biologically active, which increases water retention time, eliminates most of the contaminants including natural organic matter, transforms synthetic organic compounds, and retains pathogens from effluent water (Verma et al. 2017). Besides, trophic interactions in this layer also contribute to the bacterial removal (Haig et al. 2015b). Amongst all proposed and studied mechanisms, biological removal of contaminants has been found predominant in (mature) SSF, especially for removing pathogenic bacteria and viruses (Pfannes et al. 2015; Maurya et al. 2020). Removal of pathogenic bacteria and viruses cannot be effectively achieved by the physical straining of sand beds, as bacteria (0.1−10 µm) and viruses (0.01−0.1 µm) have small sizes compared to the pore size of SSF sand beds. A longer retention time of microbial contaminants in SSF filters (due to the slower flow rates) creates more opportunities for interactions with the indigenous microbial communities and their environment. For example, Haig et al. (2015b) firstly reported the evidence that *E. coli* removal in SSF is mainly caused by top-down trophic interactions, such as protozoan grazing and viral lysis.

Notably, the temperature and age of the Schmutzdecke were found to significantly affect the SSF removal efficiency, i.e. high temperature and matured age result in a better removal of microorganisms (Schijven et al. 2013; Haig et al. 2015a). This is consistent with the generally accepted assumption that the Schmutzdecke layer contributes most to the SSF removal efficiency, which highly associates with the activity of the complex microorganisms that inhabit the Schmutzdecke (Hijnen et al. 2004; Hijnen and Medema 2010; Pfannes et al. 2015; Schijven et al. 2013). However, viruses are considered a possible exception from the above assumption. Although some literature suggest that virus reduction could increase with Schmutzdecke maturation, most experimental data showed very limited or no virus removal in the Schmutzdecke layer (Bauer et al. 2011; Hijnen et al. 2004; Unger and Collins 2008). In the following sections, we will focus on the microbial community that has been revealed as providing the main responsibility for biological removal.

## Microbial communities in biofilters and its potential functions in biological removal

It is wildly accepted that the composition, activity, and robustness of the biological community in biofilters determine the removal effectiveness of biofiltration units in DWTPs (Basu et al. 2015; Haig et al. 2015a; Oh et al. 2018). Previous studies about the ecology of drinking water biofilters are mainly focused on quantifying microbial communities, assessing microbial diversity, and discussing the potential function of those microorganisms present in biofilters (including potential pathogens).

Regarding the community composition, bacteria are predominantly present in all DWTP biofilters (including RSF, GAC, and SSF) (Wakelin et al. 2011; Bai et al. 2013; Oh et al. 2018) (Fig. 1b). Within the bacterial community, *Proteobacteria, Planctomycetes*, *Acidobacteria, Bacteriodetes*, and *Nitrospirae* are the core community (present at all DWTPs with a mean relative abundance >1%). The phylum *Proteobacteria* has the highest relative abundance in almost all drinking water biofilters, which might be linked to the capacity of certain *Proteobacteria* to thrive in systems with low dissolved organic carbon concentrations (Gerrity et al. 2018). These bacterial groups are also found in freshwater environments and can utilise various substrates (Newton et al. 2011). The bacterial community is considered the main carbon consumer amongst the whole microbial community (Oh et al. 2018). Overall, the bioactive nature of RSF, GACF, and SSF filters has been shown to relate to nitrifiers and iron-oxidising and manganese-oxidising bacteria (Cerrato et al. 2010; Feng et al. 2012; Brandt et al. 2017; Marcantonio et al. 2020).

Although bacteria are overall predominantly present in RSF, GACF, and SSF, biofilter community dissimilarity is still found between and within DWTPs (Lautenschlager et al. 2014; Gülay et al. 2016; Palomo et al. 2016; Oh et al. 2018; Poghosyan et al. 2020). In general, differences in community composition amongst DWTPs are greater than within DWTPs. Within DWTPs, microbial communities in the filter bed share more species with their effluents than influents (Ma et al. 2020; Guarin and Pagilla 2021). Different communities developed on different materials triggered a difference between effluent communities and affected the overall quality of the effluent water. In the following, the microbial community and the corresponding (potential) functions in RSF, GACF, and SSF are reviewed in detail.

### Identity of microorganisms in biofilters and their potential functions

#### Rapid sand filtration

Bacteria were found to be the most abundant community members in RSF filters in both the filter material as well as the formed biofilms, while archaea were below 1%, and eukaryotic organisms only ranged from 4 to 7% (Table S2) (Bai et al. 2013; Gülay et al. 2016; Palomo et al. 2016).

At the phylum level, it was found that *Proteobacteria* and *Nitrospirae* were dominantly distributed in RSF filters followed by *Acidobacteria* and *Bacteriodetes* (Lautenschlager et al. 2014; Gülay et al. 2016; Palomo et al. 2016; Oh et al. 2018). The top three phyla *Proteobacteria*, *Nitrospirae*,* and Acidobacteria* build up most of the microbial community in RSF filters. Their abundance can be as high as 75−87 ± 18% of the total community (Gülay et al. 2016). The microbial community has been found with varying degrees of spatial heterogeneity in the filters (Palomo et al. 2016; Tatari et al. 2017). For instance, in a deep layer of RSF filters, *Proteobacteria* were more abundant (18.93 ± 1.31%) followed by *Nitrospirae* (9.22 ± 4.72%), while at the top of the same filters, *Nitrospirae* was found to be most abundant (26.08 ± 0.94%) followed by *Proteobacteria* (Palomo et al. 2016).

Within the microbial communities in RSF filters, the nitrifying guilds including ammonia-oxidising bacteria (AOB) (*Nitrosomonas* and *Nitrosospira*), ammonia-oxidising archaea (AOA) (*Nitrosopumilus*), and nitrite-oxidising bacteria (NOB) *Nitrobacter* and *Nitrospira* have been frequently observed and studied (Lautenschlager et al. 2014; Gülay et al. 2016; Palomo et al. 2016; Oh et al. 2018; Poghosyan et al. 2020). Tatari et al. (2017) reported a high abundance of *Nitrospira* (5−10% of the total community) consistently in RSF filters and up to 2 to 4 orders of magnitude more abundant than *Nitrobacter* and canonical AOBs, respectively. The high abundance of *Nitrospira* in nitrifying guilds is also reported by other studies (Poghosyan et al. 2020).

It was shown that the biological nitrification process is responsible for the reduction of the ammonia concentration in water in RSF filters (Oh et al. 2018; Poghosyan et al. 2020). The consistency of spatial dissimilarity of the ammonium removal capacity with the spatial heterogeneity of nitrifying guilds in RSF filters further supports the above hypothesis (Lee et al. 2014). Notably, the high abundance of *Nitrospira* spp.*,* of which some species can oxidise ammonia completely to nitrate from many examined RSF filters reveals that non-canonical pathways for nitrification may dominate the RSF filters (Tatari et al. 2017; Oh et al. 2018; Vignola et al. 2018; Poghosyan et al. 2020), although their relative contribution to NH_4_ removal in DWTPs has not been examined yet.

Distribution of microorganisms associated with the biological oxidation of iron (de Vet et al. 2011; Li et al. 2013), manganese (Cerrato et al. 2010), arsenite (As(III)) (Gude et al. 2018), sulphur (Poghosyan et al. 2020), and methane (Terry and Summers 2018; Poghosyan et al. 2020) has also been observed in RSF filters, indicating the occurrence of their corresponding biological process and possible contribution to the RSF removal performance.

#### Granular activated carbon filtration

A high abundance of bacterial genera belonging to the *Betaproteobacteria* (61–80%), *Alphaproteobacteria* (25–43%), and *Acidobacteria* (7–14%) was found in GAC filter communities (Table S4) (Magic-Knezev et al. 2008; Knezev 2015; Oh et al. 2018). Members of the genera *Bradyrhizobium* (15%)*, Rhodopseudomonas* (3.9%), and *Afipia* (2.5%), all belonging to the family *Bradyrhizobiaceae*, were dominantly present in GAC filters based on the metagenomic and 16S amplicon sequencing reported by Oh et al. (2018) and Lautenschlager et al. (2014). In addition, *Polaromonas*, *Hydrogenophaga*, *Sphingomonas*, *Methylobacterium*, and *Variovorax* were commonly isolated from the GAC filters, and their importance in biodegradation processes has been studied (Magic-Knezev et al. 2008; Wang et al. 2013; Zhou et al. 2021). *Polaromonas* organisms isolated from GACF were observed to be able to multiply at very low concentrations of carboxylic acids (Magic-Knezev et al. 2008). A *Polaromonas* strain JS666 isolated from a GACF has been observed associated with a potential utilisation of halogenated alkanes, cyclic alkanes, and (poly)aromatic compounds (Mattes et al. 2008). Members of the genus *Hydrogenophaga* can degrade methyl *tert*-butyl ether (MTBE), while members of the genus *Sphingomonas* are capable of degrading a wide range of xenobiotic compounds, including pesticides and micropollutants (i.e. terpene 2-methylisoborneol (MIB), isoproturon, polycyclic aromatic hydrocarbons (PAHs), and lindane) (Stolz et al. 2000; Kyselková et al. 2019; Abu Hasan et al. 2020). *Variovorax* organisms have been identified as bio-degraders of diverse aromatic compounds, including CECs ibuprofen (Murdoch and Hay 2015).

To further investigate the biodegradation functions of the microbial community in biofilters, Oh et al. (2018) reconstructed the metabolic pathways of biofiltration through metagenome analysis. The metabolic pathway associated with the degradation of aromatics was found to be significantly enriched in the GACF community compared to the other sand biofiltration processes. Members of the *Rhizobiales* (i.e. *Bradyrhizobium*, *Afipia*, *Rhodopseudomonas*, and *Rhizobium*) are the major groups encoding the aromatics degradation pathways amongst the total GACF community (Oh et al. 2018). It is likely that aromatics-bound DOCs are primarily biodegraded in GAC filters. The *Rhizobiales* (i.e. *Bradyrhizobium*) potentially play a key role in removing aromatic natural organic matter (NOMs) from the water during GACF (Oh et al. 2018).

#### Slow sand filtration

The microbial community of SSF filters is diverse both phylogenetically and metabolically (Haig et al. 2015a). Eukaryotes, archaea, and bacteria are all present in mature SSF filters (Table S6). Oh et al. (2018) investigated the microbial community in full-scale SSF filters with a combination of 16S rRNA gene sequencing and metagenomic analysis. The results showed that the majority of small subunit (SSU) rRNA gene sequences were phylogenetically affiliated to bacteria, while only < 1% and 2% of the SSU rRNA gene sequences were affiliated to eukaryotes and archaea, respectively.

The predominant bacterial groups have been extensively investigated (Wakelin et al. 2011; Oh et al. 2018), which are reviewed below. Members of the following bacterial phyla were abundant in various SSF filters: *Proteobacteria* (30−80%), *Actinobacteria* (1.2−16%), *Acidobacteria* (3–22%), *Planctomycetes* (4.4−14.9%), *Nitrospirae* (0−6%), and *Bacteroidetes* (4−25%) (Wakelin et al. 2011; Haig et al. 2014; Lautenschlager et al. 2014; D'Alessio et al. 2015; Li et al. 2017; Oh et al. 2018; De Souza et al. 2021). *Proteobacteria* including *Alpha-*, *Beta-*, and *Gammaproteobacteria* are always predominantly present in mature SSF filters (D’Alessio et al. 2015; Haig et al. 2014; Lautenschlager et al. 2014; Li et al. 2017; Oh et al. 2018; De Souza et al. 2021; Wakelin et al. 2011). This may be explained by the wide distribution of *Proteobacteria* bacteria in water sources and the variability of their metabolism (Newton et al. 2011; Li et al. 2017). *Alphaproteobacteria* are competitive at low nutrient concentrations (such as in river water) (Newton et al. 2011). The bacterial genus *Bradyrhizobium* belonging to the *Alphaproteobacteria* was found to be predominant in the SSF (Oh et al. 2018). They can contribute to nitrogen fixation and play a key role in removing aromatic NOMs in the SSF filter. Members of the *Betaproteobacteria* encompass a variety of methylotrophic and chemolithotrophic species, i.e. *Methylobacillus* and *Methylophilus* (D’Alessio et al. 2015). Phototrophic, chemotrophic, or chemolithotrophic bacteria belonging to the *Gammaproteobacteria* class (e.g*. Methylococcales*, *Xanthomonadales*, *Chromatiales)* were shown to be able to degrade complex organic compounds in SSF Schmutzdecke samples (Newton et al. 2011; Haig et al. 2015b).

The other predominant phyla present in SSF filters all belong to organic matter degradation-associated bacterial phyla (Liao et al. 2013b; Lautenschlager et al. 2014; D'Alessio et al. 2015; Haig et al. 2015a). For example, members of the *Actinobacteria* are commonly found in freshwater habitats where they play important roles in the degradation of organic compounds (Zhai et al. 2017). In addition, members of *Streptomyces* sp. can metabolise various compounds including sugars, amino acids, and aromatic compounds (Madigan et al. 2008). The genus *Ferruginibacter* belonging to the phylum *Bacteroidetes *has frequently been detected in water treatment plants, which are capable of hydrolysing organic matter (Zhai et al. 2017). In general, the bacterial community is the main consumer of soluble organic matter from the water (Oh et al. 2018). In addition to the above described predominant bacteria community, *Gallionella*, *Leptothrix*, and *Crenothrix* were observed and identified as the main microbial group responsible for iron and manganese oxidation in SSF (Demir 2016). The *Nitrospira* genus, belonging to nitrifying guild members, is found to be one of the dominant bacterial genera in various SSF (Oh et al. 2018; De Souza et al. 2021).

A low abundance of Archaea is observed in SSF filters (Wakelin et al. 2011; Oh et al. 2018). In a previous study, the archaeal community of the Schmutzdecke was dominated by aerobic chemo-heterotrophically *Euryarchaeota*, consisting mostly of *Halobacteriales* (photo-autotrophic taxa was excluded due to the dark environment of SSF) (Wakelin et al. 2011). Wakelin et al. (2011) hypothesised that archaea are active in the removal of dissolved organic carbon from the influent water.

Regarding the eukaryotic community, protists, protozoa, green alga, and fungi are found in SSF filters (Wakelin et al. 2011). The authors noted that the protists and protozoa are closely involved in the removal of microbial contaminants (e.g. pathogenic bacteria). This result is in line with the results found by Oh et al. (2018), who reported that the majority of the eukaryotic sequences from SSF filters were taxonomically affiliated with *Animalia* (54%) and *Viridiplantae* (20%). In particular, earthworm populations *Enchytraeidae* belonging to *Animalia* were enriched in the surface of the Schmutzdecke layer of the SSF filter. These earthworms have been found in other filter biofilms, and they can control the bacterial population by grazing (Lourenço and Nunes 2017). Notably, even at low abundance, eukaryotic communities in the SSF are strongly related to the removal of biological contaminants (e.g. through predation and grazing) (Weber-Shirk and Dick 1997; Stott et al. 2001; Haig et al. 2015b). However, a comprehensive analysis of the eukaryotic community in the Schmutzdecke of SSF is still limited.

Regarding temporal changes, fluctuations are mostly observed when a SSF is newly installed. The abundance, biodiversity, and also evenness of the microbial community in SSF filters increase with the increasing filter age (Ramond et al. 2013; Haig et al. 2015a). When the filter becomes mature, the temporal changes are marginal.

As for the spatial distribution, researchers proposed that vertical (depth) variation in the sand filter should be present in the SSF, as it relates to the chemical gradients that drive changes in community composition (Lin et al. 2012; Haig et al. 2014). However, this presumption has been challenged recently after the extensive utilisation of advanced microbial community analysis approach, i.e. 16S rRNA amplicon sequencing and metagenomics sequencing (Haig et al. 2015a; Wakelin et al. 2011; Li et al. 2017). Wakelin et al. (2011) noted that, although the highest amount of DNA content was found in the surface of the Schmutzdecke and declined along with depth, the overall community composition structure was similar through the filter depth for bacteria, archaea, and eukaryotes. Another study showed that bacterial communities of sand samples from different depths were similar in two full-scale SSFs (Haig et al. 2015a). Li et al. (2017) reported that the bacterial communities are uniform from the surface to the middle part of SSF filters with high stability. Nevertheless, further study is required to determine whether chemical gradients in water exist which might affect the SSF microbial communities (Haig et al. 2015a).

### Abiotic and biotic factors affecting the assemblage of microbial communities in biofilters

#### Abiotic factors

A significant effect of media material type on bacterial community composition in the biofilter has been observed (Gerrity et al. 2018; Oh et al. 2018; Vignola et al. 2018; Ma et al. 2020). This is due to the different properties of various filter media, such as intraparticle porosity, surface area, chemical properties, and adsorption capacity. Gerrity et al. (2018) and Vignola et al. (2018) concluded that GACF biofilter communities tend to be more phylogenetically diverse than those on other filter media types like anthracite and sand. Vignola et al. (2018) studied the development of microbial communities on sand filters and GAC, using the same source water in laboratory-scale columns. The results show that *Planctomyces* (11−13%) and *Gemmata* (1.3−8.1%) were the most abundant genera in the sand filters. In contrast, in GAC filters, the two highest relative abundance of operational taxonomic units (OTUs) on GAC filters were unidentified beyond the *Gammaproteobacteria* class level (5.6−6.2%). Species belonging to the genus of *Gordonia*, *Sulfuritalea*, and *Nitrospira* were identified as main contributors in biofiltration. They are highly abundant in sand filters but have a very low abundance in GAC filters. Significant community differences were also observed between the GAC and anthracite filters (Ma et al. 2020). In addition to filters from independent DWTPs (including studied lab-scale and pilot-scale plants), the above patterns are also consistent in different filters from the same DWTP. Lautenschlager et al. (2014) and Oh et al. (2018) studied the microbial community of biofilters including RSF, GACF, and SSF within a full-scale DWTP. The results indicated that *Bradyrhizobiaceae* were more abundant in GAC filters, whereas *Nitrospira* were enriched in the sand-associated filters (RSF and SSF).

In addition to the filter media, Oh et al. (2018) suggested that the influent water is another important factor contributing to the differences in bacterial communities of biofilters from the same DWTP. Organic carbon is removed disproportionately at each filtration stage and the GAC receives ozonated water; therefore, substances in the RSF and GACF are more easily degraded than in the SSF filter; as a result, different influent water affects the native biological community of the filter (Oh et al. 2018).

Nutrient levels such as dissolved organic carbon (DOC), ammonia nitrogen, and phosphorus in the influents could affect the bacterial diversity and community composition in biofilters (Li et al. 2010; Liao et al. 2013a; Gerrity et al. 2018; Knezev 2015). Ma et al. (2020) reported that although the influence is tempered by filter design and operating conditions (e.g. filter type, backwash) (De Souza et al. 2021), microbiomes in biofilters are primarily shaped by water quality conditions. This matches the finding of a high similarity in microbial taxa on all treatment plant filters from the same DWTPs (Lautenschlager et al. 2014). Filters operating with ozonated water generally contain significantly higher biomass levels than in the same system operating with non-ozone water. The number of different genera within the *Betaproteobacteria* was higher in GAC filters treated with ozonated water than in filters treated with non-ozone water (Knezev 2015). In addition, *Rhizobiales* were more frequently detected in GAC filters that received ozonated water. Li et al. (2010) found that the addition of phosphorus significantly changed the genus compositions of *Betaproteobacteria* in BAC reactors. The authors also found that the richness and evenness of the overall microbial community were decreased in all bioreactors when adding phosphorus. Trace amounts of organic compounds in influent water, including personal care products, household chemicals, and pharmaceutically active compounds (PhACs), have been reported to significantly alters the microbial community in biofilters. Within the Schmutzdecke, the relative abundance of *Proteobacteria* increased approximately from 30 to 99%, while the occurrence of *Bacteroidetes* dropped from 37 to 1% during the study (D’Alessio et al. 2015). These PhAC-induced microbial community changes interfered with the bacterial removal performance of SSF in Schmutzdecke, decreasing from 95% to less than 20%. Furthermore, Delgado-Gardea et al. (2019) demonstrated that additional brass in influent water changed the microbial community structure of the Schmutzdecke from a bacteria-dominated community to a eukaryote-dominated community (Delgado-Gardea et al. 2019). The brass-SSF filter had the eukaryotic *Streptophyta* dominating (31.4%), followed by *Gluconobacter* (24.6%) and *Acetobacter* family (7.7%); these genera were absent in all other SSF treatments from the same study.

RSF and GACF communities potentially select fast-growers differing from SSF communities, which is consistent with the observed highest rate of dissolved organic matter removal by RSF and GACF (Lautenschlager et al. 2014; Oh et al. 2018). This may associate with the effects of flow rates or EBCT. In other words, compared to filters operated under slow flow rates, a higher flow rate results in relatively short bed contacting time (lower EBCT) and therefore selects fast-growers in rapid filters.

In mature biofilters, the temperature had a non-significant impact on total biomass levels. However, the biological activity of the biotic community at high temperatures could be significantly higher than at low temperatures. This phenomenon is observed from season fluctuation in various biofilters. For instance, through tetrazolium reduction assays, Fonseca et al. (2001) reported that specific dehydrogenase activity was 70% higher in systems operated at ambient temperatures (> 12 °C) than in the systems held at 3 °C.

In addition to operational conditions, the biofilter scales can also affect the community composition. Drastically different responses in microbial community structure were detected in bench-scale and pilot-scale BAC reactors after phosphorus addition (Li et al. 2010). In that study, the relative abundance of perchlorate-reducing bacteria (PRB) *Dechloromonas* (the only known PRB in the system) was observed to increase from 15.2 to 54.2% in the bench-scale but decreased from 7.1 to 0.6% in the pilot-scale reactor, while *Zoogloea* increased from 17.9 to 52.0%.

#### Biotic factors

The microbial community in influent water may affect the community in filters. This effect has been frequently reported. Certain species that inhabit freshwater or influent water have a better capacity to attach and survive in biofilms of the biofilters. However, it is found that filter communities are strongly assembled by selection rather than immigration (Vignola et al. 2018). This finding is consistent with the limited effect of “bioaugmentation” in drinking water biofilters.

Bioaugmentation is an eco-friendly and economically viable method to enhance the degradation of pollutants and pathogens by adding pre-grown functional microorganisms or microbial symbionts to the media or environment. It was initially applied in wastewater treatment plants (WWTPs) and now is receiving increasing attention in DWTPs (Herrero and Stuckey 2015).

Albers et al. (2018) successfully primed nitrification in RSF filters via bioaugmentation of nitrifying communities from an existing biofilter enriched on quartz sand. Adding nitrifying community inoculum substantially decreased the lag time before nitrification commenced in the new RSF filter. However, the bioaugmented microorganisms were eventually outcompeted by native nitrifiers (Albers et al. 2018). This phenomenon is similar to the long-term loss of bioaugmented microorganisms observed in other drinking water studies (Davidson et al. 2011). It is consistent with other bioaugmented studies where a very limited effect of inoculant on the assemblage of microbial community has been found, although adding inoculant could shorten the ripening time in some biofilters (Davidson et al. 2011; Bai et al. 2016; Chan et al. 2018; Breda et al. 2019).

Several factors are hypothesised to contribute to the loss of inoculated cells from the sand filters, such as starvation due to too low levels of nutrient and assimilative organic carbon (AOC), mass-transfer limitations, antagonism by indigenous microorganisms in the filters, or simply continuous washout from the filter (Horemans et al. 2017; Albers et al. 2018). In general, the family-level composition was convergent even across different inoculants observed in other multi-replicated enrichment communities studies (Estrela et al. 2021).

### Correlation of microbial community and filtration performance

In this section, all three filtration processes are included; however, the focus will be on SSF as biofiltration plays a major role there. Although the successful function of biofiltration relies on the interaction amongst various microbial communities, especially in SSF (Wakelin et al. 2011), the correlation between the community composition and filtration efficiency (for removing chemical and biological hazards) remains unclear. Studies show that a similar filtration performance could be obtained from filters with significantly different community compositions and structures (Li et al. 2010; Vignola et al. 2018). For example, Li et al. (2010) studied the biological activated carbon (BAC) filtration in bench-scale and pilot-scale reactors operating under similar phosphorus addition conditions. After adding phosphorus, significantly different responses from the microbial community composition were observed. However, these bench and pilot scales corresponded to a similar filtering performance regarding perchlorate and nitrate removal (Li et al. 2010). In addition, Delgado-Gardea et al. (2019) reported that the bacterial removal efficiency was not significantly affected by metals, even though the brass changed the microbial community structure of the Schmutzdecke from a bacteria-dominated community to a eukaryote-dominated community (Delgado-Gardea et al. 2019).

To address this contradicting observation, researchers are trying to find out the core microbiome that is involved in biological removal processes. Some bacterial genera associated with SSF filter performance have been reported by Haig et al. (2015a). In this study, the filter performance was monitored by testing 15 water quality parameters (including ammonia, coliforms, DOC, nitrate, nitrite, total number of viable bacteria). Afterwards, the performance was further evaluated by an aggregate performance metric (Haig et al. 2014). It was found that the abundance of *Sphingomonas and* *Halomonas* (belonging to the *Proteobacteria*), and *Acinetobacter* (belonging to the *Actinobacteria)* increased during periods of better removal performance. In contrast, when the SSF filter showed poor performance, the abundance of *Acidovorax* and *Sphingobium* having similar biodegradation functions as *Sphingomonas* and *Acinetobacter* was noticed, which is explained by niche competition (Haig et al. 2015a).

From the same study, a strong positive correlation was found between species evenness and filter performance, which is in line with “the insurance hypothesis” proposed by Yachi and Loreau (1999). The hypothesis stated: “biodiversity ensures ecosystems against declines in their functioning because many species provide greater guarantees that some will maintain functioning even if others fail”. Considering that the microbial community of bioactive filters could affect and even shape the microbial community of the effluent water (Haig et al. 2014; Lautenschlager et al. 2014; Li et al. 2017; Chan et al. 2018), it seems that biostability in drinking water also benefits from a large diversity of less active species, rather than of a few very active ones. This phenomenon may only be observed in slow sand filters (i.e. SSF), while in rapid sand filters (i.e. RSF) and GACF, some stable dominant species can be more relevant because of the short reaction time.

In addition to the positive effect of communities in filters, the release of members of the microbial community from sand filters into the effluent water has been noticed (Bichai et al. 2010, 2014). If persistent pathogenic microorganisms survive in the filter or survive ingestion by protozoa, they can be released from the biofilter into the drinking water. Opportunistic pathogens were identified by 16S rRNA-based analysis, and the results suggest that the Schmutzdecke indeed contains opportunistic pathogens causing human infections (Lautenschlager et al. 2014). These findings pose a risk to drinking water safety, especially for DWTPs that use SSFs as the final treatment step. Therefore, time-course monitoring of opportunistic pathogens in the Schmutzdecke is necessary to ensure consistent drinking water quality.

## Status and challenges of the study of biofiltration in drinking water production

Approaches using descriptive and inductive measurement with correlation analysis have been widely performed in drinking water biofilter studies (Kirisits et al. 2019). As an example, the microbial community of biofilters is investigated by the sequencing of the 16S rRNA or functional genes. After grouping sequences into arbitrary OTUs, amplicon sequence variant (ASV), or bins (specific for metagenomic sequencing), statistical methods are used to identify the diversity within and amongst samples or sample groups. Afterward, quantitative correlation analysis is conducted for phylotype relative abundance and biofilter characteristics (e.g. a specific hazard group removing performance), focused on correlation expected to be statistically significant. Subsequently, induction and infer methodology are invoked to link the observed community from biofilters to the known (potential) functional communities. The resulting outcomes will be compared to literature data to find support for inference. This example is a typical top-down study approach (Faust 2019), and it remains at the descriptive level because of delivering associations rather than causal relationships.

Regarding the descriptive resolution, almost all molecular techniques including 16S rRNA and metagenome sequencing for studying microbial communities can only identify the species level but not the strain level (Prosser 2020). This limitation can lead to misleading conclusions, as the function and phenotypic characteristics can be dramatically different amongst strains from the same species. To link bio-community to specific ecological functions, bioinformatics tools such as Tax4fun (Aßhauer et al. 2015; Wemheuer et al. 2020) and PICRUST (Langille et al. 2013; Douglas et al. 2020) pipelines have been developed. The resulting 16S rRNA gene data with gene data repositories can be used to predict metagenomes (Koo et al. 2017; Li et al. 2017). For example, when ammonia oxidation is studied, the presence and amount of functional gene *amoA* encoding a subunit-A of the ammonia monooxygenase are investigated (Lee et al. 2014). However, the presence of the corresponding functional genes does not mean that these genes are expressed to contribute to the specific biodegradation/biofiltration process. In addition, the databases used by these bioinformatics tools (e.g. NCBI RefSeq database, Greengenes database) are mostly populated with medical/human-related sequences (McDonald et al. 2012; O’Leary et al. 2016; Koo et al. 2017). Therefore, for samples outside the biomedical domain, such as samples from DWTPs scenarios, inference from the default database may be not accurate.

Furthermore, to date, almost all studies on microbial communities of drinking water filters have focused on the total community via DNA-based sequencing methods. The total community (DNA) cannot distinguish the active microorganisms from dormant or dead community members, which could lead to a biased outcome. For example, significant differences between the total and active community in the drinking water distribution system (DWDS) have been noticed by Inkinen et al. (2016). In this study, the active (RNA) bacterial community consists of *Gammaproteobacteria* (*Pseudomonas* spp., *Yersinia* spp.) and *Abditibacteriota* (FBP) phyla in the biofilm from a circulating hot water system. This active community largely differed from the total community composed of mainly *Betaproteobacteria* (*Limnohabitans* sp., *Methylotenera* sp., *Comamonadaceae* family) and *Actinobacteria (Corynebacterium* spp.). Dormant or dead microorganisms do not contribute to the biodegradation/biofiltration process and so can give a biased view on the biofiltration processes. To truly understand and predict environmental processes, the distinction between active, inactive, and dead microbial cells is crucial (Singer et al. 2017). To address this issue, RNA-based techniques (such as reverse transcription (RT)-PCR) are suggested to complement DNA-based sequencing. The study of Inkinen et al. (2019) gave a more complete picture of the microbial community of biofilters by incorporating both DNA- and RNA-based sequencing methods. Furthermore, combining Stable Isotope Probing (SIP) with 16S Amplicon and metagenomics sequencing could help to further the understanding of the removal processes and the in situ trophic interactions (Haig et al. 2015b; Singer et al. 2017; Kleiner et al. 2018).

On the other hand, the bacterial group is always the main focus owing to its high abundance in the community, while the eukaryotic groups are overlooked from some studies (Lautenschlager et al. 2014; Gude et al. 2018). The importance and contribution of a microbial group should not be deduced from abundance alone, since low-abundance keystone species can play major roles in ecosystem function. A recent study of sand biofilters during manganese load fluctuations reveals that the keystone species in the ecosystem could be rare (Zhao et al. 2021). In that study, keystone microorganisms contributing to the ecological stability and Mn(II) oxidation in sand biofilters were identified by combing microbiome network and functional modules analyses. The relatively rare species *Candidatus Entotheonella palauensis* (relative abundance 0.39%) was identified as a module hub and it was presumed as one of the keystone species. In contrast, although the well-known manganese-oxidising bacterium *Hyphomicrobium* dominated the sand biofilter at all depths (relative abundance 3.3–12.7%), it exhibited only a few microbial interactions in the symbiotic network. This finding indicates that implying taxa with low abundance can play important roles in ecosystem function and should not be neglected. Regarding eukaryotic communities, even at low abundance, they may be closely correlated to the removal of biological contaminants through biological activities such as grazing or predation in the Schmutzdecke (Weber-Shirk and Dick 1997; Stott et al. 2001; Haig et al. 2015b). Therefore, a comprehensive analysis of the eukaryotic groups should be performed in future studies.

## Outlook on the microbial ecology of biofilters

To obtain a comprehensive understanding of the microbial ecology of biofiltration and promote its application to meet today's growing challenges of safe drinking water production, future studies are recommended to answer the following key questions: (i) who are the active “keystone species” in the biofilters? (ii) what bioprocess actively occurs *in-situ* during biofiltration? (iii) how does the bioprocess contribute to the filtration performance? and (iv) how to modify the assemblage of the indigenous community (through operational parameter optimisation and/or bioaugmentation, etc.) in biofilters to achieve the target biofiltration efficiency?

To address these important questions, we suggest studying both DNA and RNA in microbial community analysis. In addition, apart from the study of bacteria, eukaryotes should also be studied. We also recommend to further investigate the link of microbial diversity to specific ecological functions and reveal functional genes from the microbial community involved in metabolic and catabolic pathways. Several techniques and approaches such as meta-omics (e.g. transcriptomics analysis), nanoscale secondary ion mass spectrometry (NanoSIMS), and confocal laser scanning microscopy-fluorescent in situ hybridisation (CLSM-FISH) can be used for studying the microbial ecology of biofilters (Gebert et al. 2008; Jehmlich et al. 2016; Lueders et al. 2016; Musat et al. 2016; Singer et al. 2017; Barlow et al. 2020; Berg et al. 2020). In addition, proteomics studies and specific enzyme activity analysis are proposed to assist the functional study (Douterelo et al. 2014; Kleiner et al. 2017, 2018; Oh et al. 2018; Kumar et al. 2019; Barlow et al. 2020). Regarding the enzyme activity, the choice of the specific enzyme depends on the research question and previous findings. For example, Lautenschlager et al. (2014) observed that polysaccharides can be better degraded in the SSFs than in GAC filters and RSFs, and the authors suspected this was due to the activity of extracellular enzymes. Regarding the wish to have fewer clogging problems during biofiltration, it would be valuable to study the polysaccharide-degrading enzymes in biofilters. In addition, nitrate reductase (Nar) and nitrite reductase (Nir) were stated as the key enzymes in a biofilter that were associated with a better denitrification performance (Jia et al. 2022). Cao et al. (2022) reported that enoyl-CoA hydratase/3-hydroxyacyl-CoA dehydrogenase was strongly correlated with individual trace organic chemical transformation in the water biofiltration process.

Reviewing the current biofiltration developments in drinking water treatment systems, we observed that most studies were carried out individually, and only a few snapshots of microbial community data are available at various taxonomy levels in the specific DWTPs. To make sequencing outcomes from various studies comparable, and also understand their functional meaning in the specific ecosystem more straightforward, work similar to that of the activated sludge “MiDAS (Microbial Database of Activated Sludge)” project is proposed. Within the MiDAS project, an ecosystem-specific platform for wastewater treatment systems has been established, in which standardised wet-lab protocols and a comprehensive activated sludge microbial database of complete 16S rRNA gene sequences (MiDAS) have been discussed and developed (Dueholm et al. 2022). We believe this type of work is also valuable in drinking water ecosystems, providing a platform for generating a more comprehensive understanding of microbial ecology in drinking water production.

Furthermore, more attention should be paid on the validation and application of biofiltration research outcomes in the production of actual drinking water from a broader view. The final application aim of the biofiltration study is to develop and maintain “Good-performance” biofilters that (1) have a higher removal efficiency for target contaminants (various depending on the biofilter type, see Fig. 1c); (2) are robust to environmental fluctuation; and (3) are resistant to clogging and pathogens release problems. However, most biofiltration studies only consider one of the perspectives mentioned above. Considering the contribution of the microbial community in the biofiltration process, a comprehensive multi-dimensional model to describe the microbe community effects on all “good performance” perspectives is needed. With this model being established, a concise definition of the biofilter “healthy microbiome” may be able to be developed and be used to monitor the health status of running biofilters. For example, if the “high evenness” is included in the “healthy microbiome” definition and “keystone species” can be identified, timely monitoring of the evenness of the biofilter microbiome and detecting the abundance of the “keystone species” group via q-PCR could be included into the routine monitoring system. When the filter microbial community shows “high evenness”, and the “keystone species” can be regularly detected, the biofilter can be diagnosed as a “healthy filter”. In contrast, when the evenness and the abundance of “keystone species” are below a certain standard, an alarm should be voiced out. And the risk groups of microorganisms that may result in an unhealthy biofilter, so-called disease risky microbiome, should be further tested. In these ways, an economic and rapid solution for monitoring the “health” status of biofilters and preventing accidental outbreaks can be developed. In addition, the “pro- or prebiotics treatment” idea, regulating the filter microbial community to the status by feeding certain live microbial groups and/or nutrients, holds great promise for modulating the health status of biofilters and drinking water risk management.

To summarise, the black box of the biofiltration process in drinking water has not been illuminated yet. Future studies about “who is there?” should be conducted in the way of eliminating group biases (i.e. not only the bacteria group), and new studies answering questions such as “who is doing what?” should be focused on revealing the cause-relations between the microbial community and removal performance. Furthermore, to better apply the biofiltration research outcomes in real production, a comprehensive model describing this correlation is urgently needed. It is sincerely hoped that outcomes of biofilters studies could extensively contribute to developing safer, more reliable, and more sustainable DWTPs.

## Supplementary Information

Below is the link to the electronic supplementary material.

Supplementary file1 (PDF 314 KB)
