# Supplementary material for: Microbial ecology of biofiltration used for producing safe drinking water
Source: Appl Microbiol Biotechnol. 2022 Jun 30;106(13-16):4813–29. doi: 10.1007/s00253-022-12013-x (PMC9329406; doi:10.1007/s00253-022-12013-x)
Supplement: Supplementary file 1 — Supplementary file1 (PDF 314 KB) [file 253_2022_12013_MOESM1_ESM.pdf]

Supplementary Materials for

Title: Microbial ecology of biofiltration used for producing safe drinking water

Xi Bai[1], Inez J. T. Dinkla[2] and Gerard Muyzer[1, *]

[1]Microbial Systems Ecology, Department of Freshwater and Marine Ecology, Institute for Biodiversity and Ecosystem Dynamics, University of Amsterdam

[2]Wetsus, European Centre of Excellence for Sustainable Water Technology, Oostergoweg 9, 8911 MA Leeuwarden, The Netherlands

*Corresponding author (email: g.muijzer@uva.nl)

Number of pages: 21

Number of tables: 6

Table S1 Filtration performance of rapid sand filter.

Parameters	RSF Influent water	RSF Effluent water	Removal efficiency (% or log_{10} reduction)	Rapid Sand filter scale & DWTPs set up*	Study
Turbidity (NTU)	20.70	0.39	98.1%	DWTP: SW&GW-NA- S-***RSF***	(Bishaw and Kebede 1999)
	4−10.0 (FAU)	1−3 (FAU)	0.48 ± 0.17 log_{10} /0.62 ± 0.28 log_{10}	DWTP: SW-CS-***RSF***- Chlori (During dry/wet season)	(Asami et al. 2016)
Colour (TUC)	25	0	100%	DWTP: SW&GW-NA- S- ***RSF***	(Bishaw and Kebede 1999)
Ammonium (NH^{4+}) (mg/ L)	35	0.6−0.12	98−99.7%	Lab-scale column (corn column from DWTP: NA- PRSF- ***SRSF***) (Diameter of 2.6 cm; filter depth 5 cm)	(Tatari et al. 2016)
	0.13 ± 0.05	<0.01	>92%	DWTP: GW- aerators - Iron oxidation- PRSF- ***SRSF***	(Lee et al. 2014)
	0.1 ± 0.024	<0.02	>80%	Pilot column (Diameter of 30 cm; filter depth 70 cm)	
	0.65	0.0145	34%	DWTP: Aeration - ***PRSF***- SRSF	(Poghosyan et al. 2020)
	0.01	0.039	95%	DWTP: Aeration - PRSF- ***SRSF***	
Iron/ Fe^{2+} (mg/ L)	0.07	0.05	28.6%	DWTP: SW&GW-NA- S - ***RSF***	(Bishaw and Kebede 1999)
	0.38 ± 0.16	0.016 ± 0.048	96%	DWTP: GW- aerators - Iron oxidation- PRSF- ***SRSF***	(Lee et al. 2014)
	0.28 ± 0.18	0.009 ± 0.007	97%	Pilot column (Diameter of 30 cm; filter depth 70 cm)	
	0.11	0.053	52%	DWTP: Aeration - PRSF - ***SRSF***	(Poghosyan et al. 2020)

	0.01-0.5	<0.3	<99%	Full scale plant (7.5 × 6.2 × 3.7 m)	(Li et al. 2005)
Manganese/ Mn^{2+} (mg/L)	0.04	0.02	50%	DWTP: SW&GW-NA- S - ***RSF***	(Bishaw and Kebede 1999)
	0.035 ± 0.072	0.001 ± 0.0006	97%	DWTP: GW-aerators-Iron oxidation- PRSF-***SRSF***	(Lee et al. 2014)
	0.032 ± 0.006	0.001 ± 0.0008	97%	Pilot column (Diameter of 30 cm; filter depth 70 cm)	
	0.022	0.0079	64%	DWTP: Aeration - PRSF- ***SRSF***	(Poghosyan et al. 2020)
	0.575−3.05	<0.1	<96%	Full scale plant (7.5 × 6.2 × 3.7 m)	(Li et al. 2005)
Sulfate (mg/L)	36.74	32.5	11.6%	DWTP: SW&GW-NA- S- RSF	(Bishaw and Kebede, 1999)
Methane (mg/L)	1.2	0.012	99%	DWTP: Aeration - ***PRSF*** - SRSF	(Poghosyan et al. 2020)
Micro pesticide and Metabolites (μg/L)				DWTP: GW-PRSF-SRSF	(Hedegaard and Albrechtsen 2014)
Mecoprop (MCPP)	0.03−2.4	0.017−0.36	42−85%		
Bentazone	0.03−2.4	0.026−1.56	15−35%		
Glyphosate	0.03−2.4	0.028−2.1	7−14%		
p-Nitrophenol	0.03−2.4	0.029−2.33	1−3%		
Total bacterial (log_{10} CFU/L)	-	-	17−50% (min−average)	Full scale water treatment plant	(O'Connor and O'Connor 2001)
Faecal coliform (MPN/0.1L)	230	0	100%	DWTP: SW&GW-NA- S- ***RSF***	(Bishaw and Kebede, 1999)

Total coliforms (log_{10} CFU/L)	3.02−4.14	1.30−3.79	0.36 ± 0.66 log_{10}/1.24 ± 0.87 log_{10}	DWTP: SW-CS-***RSF*** -Chlori (During dry/wet season)	(Asami et al. 2016)
E. coli (log_{10} CFU/L)	2.3−3.13	<1−2.92	0.06 ± 0.36 log_{10}/ 0.59 ± 0.50 log_{10}	DWTP: SW-CS-***RSF*** -Chlori (During dry/wet season)	(Asami et al., 2016)
Cryptosporidium parvum oocysts	-	-	1.7−3 log_{10}	Pilot plant: CS-***RSF***-GACF-Chlori (Sand column: diameter 17cm, length 1.6 m)	(Gitis 2008)
PMMoV	-	-	0.78 log_{10} /1.26 ± 0.25 log_{10}	DWTP: SW-CS -***RSF*** -Chlori (During dry/wet season)	(Asami et al., 2016)
JC PyV	-	-	0.59 log_{10} / 0.49± 0.57 log_{10}	DWTP: SW-CS -***RSF*** -Chlori (During dry/wet season)	(Asami et al., 2016)

*****SW: surface water; GW: ground water; (C)S: (Coagulation and) sedimentation; Chlori: chlorination; PRSF: primary RSF; SRSF: secondary RSF; NA: unknown.

Table S2 Microbial communities of rapid sand filters.

Study	Research method	Characterisations of RSF	Sampling position	Microbial community	Relative abundance or Major genera
Lautenschlager et al. 2014	16S rRNA gene-based 454 pyrosequencing analysis	Full-scale RSF (DWTP consist of pre-ozonation, RSF, intermediate ozonation, GACF, SSF)	Filter surface	<u>*Proteobacteria*,</u> *Planctomycetes*, *Acidobacteria*, *Bacteroidetes*, *Nitrospira*, and *Chloroflexi*	*Nitrospira* (8.1%)
Oh et al. 2018	Metagenomic analysis	Full-scale RSF (DWTP consist of pre-ozonation, RSF, intermediate ozonation, GACF, SSF)	Filter surface	<u>*Proteobacteria*,</u> *Bacteroidetes*, *Firmicutes*, and *Actinobacteria*	*Nitrospira* (5.1%); *Polaromonas* (2.0%); *Burkholderia* (2.0%); *Bradyrhizobium* (1.8%)
Gude et al. 2018	16S rRNA (V3-V4)	Pilot-scale filter sand columns fed with drinking water with spikes of As (III)	-	*Proteobacteria*, *Actinobacteria*, *Nitrospira*, and others	-
Poghosyan et al., 2020	High-throughput sequencing	RSF from full-scale DWTP, consist of PRSF and SRSF*****	-	*Gammaproteobacteria* (17-67%), *Bacteroidota* (5-17%), *Acidobacteria* (2-15%), *Alphaproteobacteria* (4-12%), *Planctomycetota* (2-10%), *Ca. Patescibacteria* (CPR: 1-7%), and *Nitrospirota* (2-6%)	-

*****PRSF: primary RSF; SRSF: secondary RSF. Microorganism groups present in high abundance are highlighted with an underscore.

Table S3 Filtration performance of granular activated carbon filter.

Parameters	GACF Influent water	GACF Effluent water	Removal efficiency (% or log_{10} reduction)	Filter scale & Set-up	Study
Turbidity (NTU)	5.70	0.50	91%	Pilot filtration plant used to treat stormwater; Slow GAC filters	(Wakelin et al. 2010)
Colour (HU)	10.00	5.30	47%	Pilot filtration plant used to treat stormwater; Slow GAC filters	(Wakelin et al. 2010)
DOC	-	-	3−10%	Bench-scale columns (internal diameter 0.0375; depth 0.4m; GAC (2 years old): diameter at 1.0-1.2 mm) (EBCT of 10 min and 18 min)	(Zhang et al. 2017)
	-	-	11%−14% / 15%−20%	Pilot GAC filters (EBCT of 8 min /16 min)	(Thiel et al. 2006)
TOC (mg/L)	-	-	9%	NA	(Chowdhury et al. 2010)
	4.70	2.20	53%	Pilot filtration plant used to treat stormwater; Slow GAC filters	(Wakelin et al., 2010)
	-	-	22%	Pilot filtration plant: SW-PF-OZ- GACF-UF	(Velten et al. 2011)
AOC (μg C/L)	305	105	65.6%	At 20 drinking water treatment works	(Pick et al. 2019)
	-	-	74 ±8%	Pilot GAC filters (1.22m GAC (top) + 0.30 sand (bottom))	(Greenstein et al. 2018)

	-	-	39 ± 1%	Pilot GAC filters (1.22m GAC (top) + 0.30 sand (bottom))	(de Vera et al. 2019)
Ammonium (NH^{4+}) (mg/ L)	0.07	0.006	91.4%	Pilot filtration plant used to treat stormwater; Slow GAC filters	(Wakelin et al., 2010)
Total coliforms (log_{10} CFU/L)	4.76	3.31	1.45 log_{10}	Pilot filtration plant used to treat stormwater; Slow GAC filters	(Wakelin et al., 2010)
E. coli (log_{10} CFU/L)	-	-	0–0.1 log_{10} / 0.3 log_{10}	Pilot plant filters (diameter 0.15 m; depth 1 m; grain size 0.8-1.1 mm) (with fresh /loaded GAC)	(Hijnen et al. 2010)
	3.11	<1	>2.11 log_{10}	Pilot filtration plant used to treat stormwater; Slow GAC filters	(Wakelin et al., 2010)
Clostridium bifermentans/Spores of Sulphite- Reducing Clostridia (SSRC)	-	-	0.4–0.6 log_{10} / 0.4 log_{10}	Pilot plant filters (diameter 0.15 m; depth 1 m; grain size 0.8-1.1 mm) (with fresh/loaded GAC)	(Hijnen et al., 2010)
Cryptosporidium (Log_{10} oocysts/L)	5.20	4	1.2 log_{10}	Column filter (height h 1 m, porosity 0.32; grain size d 0.65 mm)	(Bichai et al. 2014)
	-	-	2.70 log_{10} / 1.20 log_{10}	Pilot plant filters (diameter 0.15 m; depth 1 m; grain size 0.8-1.1 mm) (with fresh/loaded GAC)	(Hijnen et al., 2010)
	-	-	32.1% / 66.2%	Pilot plant filters (diameter 0.15 m; height 1.0 m, (EBCT of 12 min)(in the lower / upper parts of the GAC filter beds)	(Bichai et al. 2010)
Giardia (Log_{10} cysts/L)	4.65	2.70	2.1 log_{10}	Column filters(height h 1 m, porosity 0.32; grain size d 0.65 mm)	(Bichai et al., 2014)

	-	-	2.0−2.1 $\log_{10}$ / 2.1−2.2 $\log_{10}$	Pilot plant filters (diameter 0.15 m; depth 1 m; grain size 0.8-1.1 mm) (with fresh/loaded GAC)	(Hijnen et al., 2010)
Contaminants of emerging concern (CECs)					
Acetaminophen, Ibuprofen, Trimethoprim, Aminotriazole, and 17b- estradiol			>80%,	Bench-scale columns (internal diameter 0.0375; depth 0.4m) (GAC (2 years old): diameter at 1.0-1.2 mm) (EBCT of 10 min/18 min)	(Zhang et al. 2017)
tris(2-carboxyethyl)phosphine (TCEP), cotinine, and iopromide			22%-46%/ 59%-84%	Bench-scale columns (internal diameter 0.0375; depth 0.4m) (GAC (2 years old): diameter at 1.0-1.2 mm) (EBCT of 10 min/18 min)	(Zhang et al. 2017)
16 CECs (including acetaminophen and ibuprofen; erythro-mycin, sulfamethoxazole, and trimethoprim; carba-mazepine; atenolol; gemfibrozil; TCEP; cotinine; aminotriazole, atrazine, and DEET; caffeine; 17b-estradiol and, iopromide			>75%	Bench-scale columns (internal diameter 0.0375; depth 0.4m) (GAC (2 years old): diameter at 1.0-1.2 mm; with a pre-ozonation dosage of 3 mg/L) (EBCT of 18 min)	(Zhang et al. 2017)

SW: surface water; PF: prefiltration; OZ: ozonation; UF; ultrafiltration; NA: unknow

Table S4 Microbial communities of granular activated carbon filters.

**Study**	**Research method**	**Characterisations of SSF**	**Sampling position**	**Dominant Microbial phyla/community (Relative abundance)**	**Major class and/or genera (Relative abundance)**
Lautenschlager et al., 2014	16S rRNA gene-based 454 pyrosequencing analysis	Full-scale SSF (DWTP consist of pre-ozonation, RSF, intermediate ozonation, GACF, SSF)	Filter surface (directly after backwashing)	*Proteobacteria* (74.2%), *Acidobacteria*, *Bacteriodetes*, *Nitrospira* (3.3%), *Chloroflex*, *Gemmatimonadetes*	*Rhizobiales*
Oh et al., 2018	Metagenomic analysis		Filter surface	*Betaproteobacteria*, *Alphaproteobacteria*, *Acidobacteria*	*Bradyrhizobium* (15,3%); *Rhodopseudomonas* and *Afipia* belonging to *Bradyrhizobiaceae*
Knezev 2015	16S rRNA gene-targeted T-RFLP fingerprinting (8F-FAM - 1392R)	Full-scale and Pilot-plant filters	Filter beds	*Betaproteobacteria* (43% of sequences, 31% of OTUs), *Alphaproteobacteria* (38%, 36%), *Acidobacteria* (7%, 9%)	*Comamonadaceae*, *Hyphomicrobiaceae*, *Rhodobacteriaceae*, and *Bradyrhizobiaceae*

Microorganisms groups present in high abundance are highlighted with an underscore.

Table S5 Filtration performance of slow sand filters.

Parameters	SSF Influent water	SSF Effluent water	Removal efficiency (% or log10 reduction)	Filter scale & Set-up	Study
Turbidity (NTU)	2.711	-	27.24%−39.18%	Lab-scale-column (length: 0.96 m; diameter 0.3 m; sand particle size 0.5 mm, flow rate 0.04−0.40 m/h)	(Bellamy et al. 1985)
	-	-	94.8 ±2.80 %−98,2 ±0.79 %	Lab-scale column (length: 0.375m; surface 0.24 m^2 sand particle size 0,1 mm)	(Demir 2016)
	15.62 ± 10.1	1.24 ± 0.53	89.0% ± 6.9 %	Household column (depth: 0.6 m; diameter: 0.305 m; Sand d_{10}= 0.17−0.52 mm)	(Jenkins et al. 2011)
Colour (HU)	10.00	8.00	20%	Lab-scale-column (length: 0.67 m; diameter 0.063 m; sand particle size 0.2 mm)	(Wakelin et al., 2010)
Total dissolved solids (mg/L)	83.00	132.00	59%		
Total organic carbon (mg/L)	4.70	4.40	6.4%		
Total nitrogen (mg/L)	0.56	0.88	-57.14%		
Nitrate + nitrite (mg/L)	0.12	0.56	-44%		
Nitrate nitrogen (NO3–N) (mg/L)	-	-	94%−99%	Lab-scale-column (length: 1 m; diameter 0.13 m; sand particle size 0.5 mm) (removal measured at 80 cm filter depth)	(Aslan and Cakici 2007)
Ammonium (NH_4^+) (mg/L)	0.07	0.03	57%	Lab-scale-column (length: 0.67m; diameter 0.063m, sand particle size 0.2 mm)	(Wakelin et al., 2010)

Iron/ Fe_2^+ (mg/l), Manganese/ Mn_2^+ (mg/L)	-	< 0.1	90.4±4.80% −95.4±4.8%	Lab-scale column (length: 0.375m; surface 0.24 m^2; sand particle size 0.1 mm)	(Demir 2016)
Pharmaceutically active compounds					
○ 17-β estradiol (µg/L)	50	-	21%−56%	Lab-scale column (length: 0.88 m; surface 0.24 m^2; sand 0.10 mm particle size)	(D'Alessio et al. 2015)
○ caffeine (µg/L)	50	-	100%		
○ carbamazepine (µg/L)	50	-	<10%		
○ estrone (µg/L)	50	-	11%−88%		
○ gemfibrozil (µg/L)	50	-	<10%		
○ phenazone (µg/L)	50	-	<10%		
Giardia (Log_{10} oocysts/L)	1.7−3.71	-	99.981%−99.994%	Lab-scale-column (length: 0.96 m; diameter 0.3 m; sand particle size 0.5 mm)	(Bellamy et al. 1985)
	4.51±3.7	-2.1	6.61−7.03	Pilot-filter (length: 1.29 m; surface 2.56 m^2; diameter 0.05 m; sand d_{50}=0.61 mm; flow rate 0.45 m/h)	
Phytophthora cryptogea Zoospores (Log_{10} CFU/L)	2.56	-	100%	Lab-scale-column (length: 1 m; diameter 0.16 m; sand 0.30 mm particle size; SSF runs for more than 21 days)	(Calvo-Bado et al. 2003)

Oocysts of *Campylobacter*	3−5.23	-	>5−6 log_{10}	Lab-scale-column (length: 0.4 m; diameter 0.09 m; ripen sands; influent water pre-treated with coagulation, rapid sand filtration, softening, etc.)	(Hijnen et al. 2004b)
Campylobacter (Log_{10} CFU/mL)	-	-	3.4 ± 0.6 log_{10}	Full-scale filters (Influent water pre-treated with coagulation, rapid sand filtration, softening, etc.)	(Hijnen et al. 2004b)
Thermotolerant coliforms (Coli44) (Log_{10} CFU/L)	-	-	2.6 ± 0.5 log_{10}	Full-scale filters (Influent water pre-treated with coagulation, rapid sand filtration, softening, etc.)	(Hijnen et al. 2004b)
Total coliforms (Log_{10} CFU/mL)	-	-	99.67% −99.96%	Lab-scale-column (length: 0.96 m; diameter 0.3 m; sand particle size 0.5 mm)	(Bellamy et al. 1985)
	4.76	1.70	3.06 log_{10}	Lab-scale-column (length: 0.67 m; diameter 0.063 m; sand particle size 0.2 mm)	(Wakelin et al. 2010)
	3.97−4.35	-	2−4.7 log_{10}	Lab-column (length: 0.5 m; diameter 0.05 m; flow rate 0.03 m/h) (sand d_{10}= 0.07 mm)	(Yogafanny et al. 2014)
	3.97−4.35	-	1.6−2.5 log_{10}	Lab-column (length: 0.5 m; diameter 0.05 m; flow rate 0.03 m/h) (sand d_{10}= 0.13mm)	
	3.97−4.35	-	2.7 log_{10}	Lab-column (length: 0.5 m; diameter 0.05 m; flow rate 0.03 m/h) (sand d_{10}= 0.2 mm)	
	3.04	0.61−1.89	99.29%−99.96%	Lab-scale-column (sand particle size 0.15-0.595 mm; flow rate 0.2 m/h)	(matuzahroh et al. 2020)
	3.04	0.63−2.04	99.00%−99.96%	Lab-scale-column (sand particle size 0.15−0.595 mm; flow rate 0.4 m/h)	
	3.97−4.35	-	1.6−4.7	Lab-column (length: 0.5 m; diameter 0.05 m; sand d10= 0.07-0.2 mm; flow rate 0.03 m/h)	(Yogafanny et al. 2014)
	-	-	10% / 80%	Pilot scale filter (0.20 m3, 57 cm inner diameter, 88 cm height) (3 PhACs spiking events occurred.) (Schmutzdecke layer / whole SSF filter)	(D'Alessio et al. 2015)

Faecal coliform (log_{10} CFU/ mL)	-	-	98.45%−99.84%	Lab-scale-column (length: 0.96 m; diameter 0.3 m; sand particle size 0.5 mm)	(Bellamy et al. 1985)
	3.17 ± 0.48	-	1.4 ±0.4 log_{10}	Household column （depth: 0.6m; diameter:0.305 m; Sand d_{10}=0.17-0.52 mm）	(Jenkins et al., 2011)
E. coli (log_{10} CFU/mL)	-	-	4.11 log_{10}	Pilot-scale（Depth: 0.9m; surface:49-90 m^2; Sand d_{10}= 0.269 mm）	(Bauer et al. 2011)
	3.11	<1	>2.11 log_{10}	Lab-scale-column (length: 0.67m; diameter 0.063m; sand particle size 0.2 mm)	(Wakelin et al., 2010)
	2.55 ± 0.33−2.71 ± 0.44		0.4−4.3 log_{10}	Lab-scale-column (length: 0.4 m; 60L; sand d10= 0.19-0.22 mm; flow rate 0.9 L/min)	(Elliott et al. 2008)
	-	-	2−3log_{10}	Full-scale and Pilot-scale (Depth: 1.5m; surface:2.56 m^2; 0.3 mm diameter sand	(Hijnen et al. 2004b)
	3.59−3.96		1.6 – 5 log_{10}	Lab-column (length: 0.5 m; diameter 0.05 m; sand d10= 0.07-0.2 mm; flow rate 0.03 m/h)	(Yogafanny et al. 2014)
			15% / 85%	Pilot scale filter (0.20 m3, 57 cm inner diameter, 88 cm height) (3 PhACs spiking events occurred.) (Schmutzdecke layer / whole SSF filter)	(D'Alessio et al. 2015)
Intestinal *enterococci* (log_{10} CFU/mL)	-	-	3.65 log_{10}	Pilot-scale (depth: 0.9 m; surface:49-90 m^2; Sand d_{10}= 0.269 mm）	(Bauer et al. 2011)
Echovirus type 12	2.90 ± 0.17 −2.48 ± 0.62	-	1.14−>2 log_{10}	Lab-scale-column (length: 0.4 m; 60L; sand d10= 0.19-0.22 mm; flow rate 0.9 L/min)	(Elliott et al. 2008)
K13-phages (log_{10} PFU/ mL)	-	-	2.45 log_{10} / >4.23 log_{10}	Pond-scale (surface: 23 m^2; Sand d_{10}= 0.269 mm) (depth: 0.3m/1.2m)	(Bauer et al. 2011)

	-	-	3.58 log_{10}	Pilot-scale (surface: 49-90 m^2; Sand d_{10}=0.269 mm; depth: 0.9m）	
MS2 (log_{10} PFU/ mL)	-	-	1.5−2 log_{10}	Full-scale and Pilot-scale (Depth: 1.5m; surface:2.56 m^2; 0.3 mm diameter sand	(Hijnen et al. 2004b)
	3.48 ±1.14		0.54 ±0.42 log_{10}	Household column (depth: 0.6m; diameter:0.305m; Sand d10= 0.17−0.52 mm）	(Jenkins et al. 2011)
	-	-	0.2−2,2 log_{10}	Pilot plants: RW-OZ-RF-GAC-SSF(0.45m)	(Anderson et al. 2009)
	-	-	99%	Pilot-scale (depth: six feet; surface:4.6 m^2）	(Yahya et al. 1993)
	2.74 ± 1.01 − 3.10 ± 0.25	-	0.061 − >1 log_{10}	Lab-scale-column (length: 0.4 m; 60L; sand d10= 0.19-0.22 mm; flow rate 0.9 L/min)	(Elliott et al. 2008)
PRD-1 (log_{10} PFU/ mL)	3.68 ± 0.18 − 3.50 ± 0.36	-	0.053 − >1 log_{10}	Lab-scale-column (length: 0.4 m; 60L; sand d10= 0.19 − 0.22 mm; flow rate 0.9 L/min)	(Elliott et al. 2008)
Somatic phage (log_{10} PFU/ mL)	-	-	1.47 log_{10} / 3.07 log_{10}	Pond-scale (surface: 23 m^2; Sand d_{10}= 0.269 mm) (depth: 0.3m/1.2m)	(Bauer et al. 2011)
	-	-	2.74 log_{10}	Pilot-scale (surface: 49-90 m^2; Sand d_{10}=0.269 mm; depth: 0.9m）	
Spores of Sulphite-Reducing Clostridia (SSRC)	-	-	1.4-4.2 log_{10}	6 different DWTPs	(Hijnen et al. 2004a)
	-	-	2-3 log_{10}	Lab-scale-column (length: 0.4 m; diameter 0.09 m; ripen sands; influent water pre-treated with coagulation, rapid sand filtration, softening, etc.)	(Hijnen et al. 2004b)
P. cryptogea Zoospores (CFU/L)	359-399	0	100%	Lab-scale-column (length: 1 m; diameter 0.16 m; sand 0.30 mm particle size; SSF runs for more than 21 days)	(Calvo-Bado et al. 2003)
AdV-Genomes	-	-	1.47 log_{10} / >1.88 log_{10}	Pond-scale (surface: 23 m^2; Sand d_{10}= 0.269 mm) (depth: 0.3m/1.2m)	(Bauer et al. 2011)

	-	-	> 2.88 log_{10}	Pilot-scale (surface: 49-90 m^2; Sand d_{10}=0.269 mm; depth: 0.9m)

Table S6 Microbial communities of slow sand filters.

Study	Research method	Characterisations of SSF	Sampling position	Dominant Microbial phyla/community (Relative abundance)	Major class and/or genera (Relative abundance)
Lautenschlager et al., 2014	16S rRNA gene-based 454 pyrosequencing analysis	Full-scale SSF (DWTP consist of pre-ozonation, RSF, intermediate ozonation, GACF, SSF)	Filter surface	*Proteobacteria*, *Acidobacteria*, *Planctomycetes*, *Chloroflexi*, *Actinobacteria*, *Chlorobi*, *Bacteriodetes*, *Nitrospirae* (5.6%)	-
Oh et al., 2018	Metagenomic analysis (encode prokaryotic SSU rRNA)		Filter surface (including Schmutzdecke)	*Proteobacteria*, *Bacteroidetes*, *Firmicutes*, and *Actinobacteria*	*Nitrospira* (5.6%); *Solibacter* (1.8 %); *Gemmatimonas* (1.75 %); *Pseudomonas* (1.65 %); *Burkholderia* (1.7 %); *Bradyrhizobium* (1.6%); *Planctomyces* (1.45%)
	Metagenomic analysis (encode eukaryotic SSU rRNA)		Schmutzdecke	*Animalia* (54%) (including phylum: *Annelida* (23%), *Arthropoda* (16%), *Mollusca* (3%), *Nematoda* (5%), *Platyhelminthes* (5%), and *Rotifera* (1%)) *Viridiplantae* (20%)	*Enchytraeidae* (70% of *Annelida* sequencing)

Haig et al. 2014	16S rRNA gene-based 454 pyrosequencing analysis (515F - 926R)	Full- scale SSFs & Lab-scale columns	Depths 0, 4, 10, 15, 20, 30, 40 and 50 cm	*Proteobacteria* (60.80%), *Bacteriodetes* (9.27%), *Acidobacteria* (5.17%), *Actinobacteria* (4.67%), *Planctomycetes* (4.42%), *Verrucomicrobia* (1.78%), *Gemmatimonadetes* (1.15%)	*Alphaproteobacteria*, *Betaproteobacteria*
Wakelin et al., 2011	18S rRNA gene sequencing (82FE-1391RE)	Lab-scale column (180 cm long, 6 cm diameter)	Depths 0, 5, 20 and 45 cm	*Ciliophora* (13%), *Viridiplantae*, *Cercozoa*, *Stramenopile*, *Amoebozoa*, *Aschomycota*, and *Rozellida*	*Cercozoa* (Ebridd-type protists); Ciliate protozoa, green microalgae, stramenopiles, amoeboid protozoa and fungi
	16S rRNA sequencing (A109f-A934b)			*Euryarchaeota*	*Halobacteriales*
	High-density microarray (27F - 1492R)			*Proteobacteria* (43%); *Firmicutes* (24%) and *Actinobacteria* (16%)	*Alphaproteobacteria* (16%), *Clostridia* (16%), *Actinobacteria* (16%), *Betaproteobacteria* (14%), *Gammaproteobacteria* (9%), *Planctomycetes* (4%), *Chloroflexi* (4%)
(De Souza et al. 2021)	16S rRNA sequencing (341F -806R)	Community-scale Conventional Slow Sand Filter	Depth 0, 5, 20, and 30 cm.	*Proteobacteria* (42%–80%), *Acidobacteria* (3%–22%), *Verrucomicrobia* (5%–16%), *Chloroflexi* (3%–15%), *Bacteroidetes* (4%–12%), *Actinobacteria* (2%-6%), *Nitrospirae* (0%–6%), *Chlorobi* (1%–6%), and *Cyanobacteria* (0%–2%)	*Geobacter* (1%–23%), *Nitrospira* (1%–9%), *Anaeromyxobacter* (0%–8%), *Hyphomicrobium* (1%–10%), *Candidatus Solibacter* (0%–9%), *Rhodoplanes* (1%–6%), *Mycobacterium* (0%–6%), and *Chthoniobacter* (0%–6%)

D'Alessio et al., 2015	16S rRNA sequencing (515F -806R)	Lab-scale SSF barrels (0.88 m depth, 0.57 m diameter, sand (d_{10} = 0.30 mm)	Schmutzdecke	*Proteobacteria*, *Bacteroidetes*	*Gammaproteobacteria* [10–99%]
			Depth 5, 10, 20cm.	*Proteobacteria* (35–52%), *Bacteroidetes* (13–25%), *Acidobacteria* (7–17%)	Alpha-, Beta-, Delta-, and *Gammaproteobacteria*
Delgado-Gardea et al. 2019	16S rRNA sequencing	Lab-scale SSF column (0.6m depth, 0.2 m diameter, 0.4 mm fine sand)	Schmutzdecke	*Proteobacteria* (42.89%), *Acidobacteria* (16.92%), *Planctomycetes* (14.9), *Bacteroidetes* (6.46%), *Verrucomicrobia* (4.38%), *Actinobacteria* (1.2%), *Firmicutes* (0.7%), *Cyanobacteria* (1.3%)	*Chloracidobacterium* (4.7%), *Comanonadaceae* (4.6%), *Kaistobacter* (1.7%), *Planctomyces* (7.8), *Gemmata* (1.7), *Nitrospira* (2.1%)
(Li et al. 2019)	16S rRNA sequencing (V4 region)	Lab-scale GAC-SSF sandwich column	Top of the SSF	*Proteobacteria* (>50%), *Bacteroid*etes, *Actinobacteria*, *Verrucomicrobia*, *Planctomycetes*, *Patescibacteria*, *Cyanobacteria*.	*Gammaproteobacteria*

Microorganism groups present in high abundance are highlighted with an underscore.

Reference

Anderson WB, Deloyde JL, Van Dyke MI, Huck PM (2009) Influence of design and operating conditions on the removal of MS2 bacteriophage by pilot-scale multistage slow sand filtration. J Water Supply Res Technol - AQUA 58:450–462. doi:10.2166/aqua.2009.140

Asami T, Katayama H, Torrey JR, Visvanathan C, Furumai H (2016) Evaluation of virus removal efficiency of coagulation-sedimentation and rapid sand filtration processes in a drinking water treatment plant in Bangkok, Thailand. Water Res 101:84–94. doi:10.1016/j.watres.2016.05.012

Aslan S, Cakici H (2007) Biological denitrification of drinking water in a slow sand filter. J Hazard Mater 148:253–258. doi:10.1016/j.jhazmat.2007.02.012

Bauer R, Dizer H, Graeber I, Rosenwinkel KH, López-Pila JM (2011) Removal of bacterial fecal indicators, coliphages and enteric adenoviruses from waters with high fecal pollution by slow sand filtration. Water Res 45:439–452. doi:10.1016/j.watres.2010.08.047

Bellamy WD, Silverman GP, Hendricks DW, Logsdon GS (1985) Removing *Giardia* cysts with slow sand filtration. J Am WATER Work Assoc77:52–60. doi: 10.1002/j.1551-8833.1985.tb05492.x

Bichai F, Barbeau B, Dullemont Y, Hijnen W (2010) Role of predation by zooplankton in transport and fate of protozoan (oo)cysts in granular activated carbon filtration. Water Res 44:1072–1081. doi:10.1016/j.watres.2009.09.001

Bichai F, Dullemont Y, Hijnen W, Barbeau B (2014) Predation and transport of persistent pathogens in GAC and slow sand filters: a threat to drinking water safety? Water Res 64:296–308. doi:10.1016/j.watres.2014.07.005

Bishaw D, Kebede F (1999) Evaluation on the efficiency of rapid sand filtration. Integrated development for water supply and sanitation: proceedings of the 25th WEDC Conference, Ethiopia.

Calvo-Bado LA, Pettitt TR, Parsons N, Petch GM, Morgan JAW, Whipps JM (2003) Spatial and temporal analysis of the microbial community in slow sand filters used for treating horticultural irrigation water. Appl Environ Microbiol 69:2116–2125. doi:10.1128/AEM.69.4.2116-2125.2003

Chowdhury Z, Traviglia A, Carter J, Brown T, Summers RS, Corwin C, Zearley T, Thurman M, Ferrara I, Olson J, Barron P (2010) Cost-effective regulatory compliance with GAC biofilters. Water Research Foundation Report 4155, Denver, CO

O’Connor JT, O’Connor TL (2001) Removal of Microorganisms by Rapid Sand Filtration. H2O'C Engineering, Columbia, pp 23-35.

D'Alessio M, Yoneyama B, Kirs M, Kisand V, Ray C (2015) Pharmaceutically active compounds: their removal during slow sand filtration and their impact on slow sand filtration bacterial removal. Sci Total Environ 524–525:124–135. doi:10.1016/j.scitotenv.2015.04.014

de Vera GA, Lauderdale C, Alito CL, Hooper J, Wert EC (2019) Using upstream oxidants to minimize surface biofouling and improve hydraulic performance in GAC biofilters. Water Res 148:526–534. doi:10.1016/j.watres.2018.10.085

Delgado-Gardea MCE, Tamez-Guerra P, Gomez-Flores R, Garfio-Aguirre M, Rocha-Gutiérrez BA, Romo-Sáenz CI, Zavala-Díaz de la Serna FJ, Eroza-de la Vega G, Sánchez-Ramírez B, González-Horta M del C, Infante-Ramírez M del R (2019) Streptophyta and acetic acid bacteria succession promoted by brass in slow sand filter system Shmutzdeckes. Sci Rep 9:7021. doi:10.1038/s41598-019-43489-9

Demir NM (2016) Experimental study of factors that affect iron and manganese removal in slow sand filters and identification of responsible microbial species. Polish J Environ Stud 25:1453–1465. doi:10.15244/pjoes/62679

Elliott MA, Stauber CE, Koksal F, DiGiano FA, Sobsey MD (2008) Reductions of *E. coli*, echovirus type 12 and bacteriophages in an intermittently operated household-scale slow sand filter. Water Res 42:2662–2670. doi:10.1016/j.watres.2008.01.016

Gitis V (2008) Rapid sand filtration of *Cryptosporidium parvum*: effects of media depth and coagulation. Water Sci Technol Water Supply 8:129–134. doi:10.2166/ws.2008.058

Greenstein KE, Lew J, Dickenson ERV, Wert EC (2018) Investigation of biotransformation, sorption, and desorption of multiple chemical contaminants in pilot-scale drinking water biofilters.

Chemosphere 200:248–256. doi:10.1016/j.chemosphere.2018.02.107

Gude JCJ, Rietveld LC, van Halem D (2018) Biological As(III) oxidation in rapid sand filters. J Water Process Eng 21:107–115. doi:10.1016/j.jwpe.2017.12.003

Haig SJ, Quince C, Davies RL, Dorea CC, Collins G (2014) Replicating the microbial community and water quality performance of full-scale slow sand filters in laboratory-scale filters. Water Res 61:141–151. doi:10.1016/j.watres.2014.05.008

Hedegaard MJ, Albrechtsen HJ (2014) Microbial pesticide removal in rapid sand filters for drinking water treatment - Potential and kinetics. Water Res 48:71–81. doi:10.1016/j.watres.2013.09.024

Hijnen WAM, Medema GJ, Van Der Kooij D (2004a) Quantitative assessment of the removal of indicator bacteria in full-scale treatment plants. Water Sci Technol Water Supply 4:47–54. doi:10.2166/ws.2004.0027

Hijnen WAM, Schijven JF, Bonné P, Visser A, Medema GJ (2004b) Elimination of viruses, bacteria and protozoan oocysts by slow sand filtration. Water Sci Technol 50:147–154. doi:10.2166/wst.2004.0044

Hijnen WAM, Suylen GMH, Bahlman JA, Brouwer-Hanzens A, Medema GJ (2010) GAC adsorption filters as barriers for viruses, bacteria and protozoan (oo)cysts in water treatment. Water Res 44:1224–1234. doi:10.1016/j.watres.2009.10.011

Jenkins MW, Tiwari SK, Darby J (2011) Bacterial, viral and turbidity removal by intermittent slow sand filtration for household use in developing countries: experimental investigation and modeling. Water Res 45:6227–6239. doi:10.1016/j.watres.2011.09.022

Knezev A (2015) Microbial activity in granular activated carbon filters in drinking water treatment. Doctoral dissertation, Wageningen University and Research

Lautenschlager K, Hwang C, Ling F, Liu WT, Boon N, Köster O, Egli T, Hammes F (2014) Abundance and composition of indigenous bacterial communities in a multi-step biofiltration-based drinking water treatment plant. Water Res 62:40–52. doi:10.1016/j.watres.2014.05.035

Lee CO, Boe-Hansen R, Musovic S, Smets B, Albrechtsen HJ, Binning P (2014) Effects of dynamic operating conditions on nitrification in biological rapid sand filters for drinking water treatment. Water Res 64:226–236. doi:10.1016/j.watres.2014.07.001

Li D, Zhang J, Wang H, Yang H, Wang B (2005) Operational performance of biological treatment plant for iron and manganese removal. J Water Supply Res Technol - AQUA 54:15–24. doi:10.2166/aqua.2005.0002

Li J, Han X, Brandt BW, Zhou Q, Ciric L, Campos LC (2019) Physico-chemical and biological aspects of a serially connected lab-scale constructed wetland-stabilization tank-GAC slow sand filtration system during removal of selected PPCPs. Chem Eng J 369:1109–1118. doi:10.1016/j.cej.2019.03.105

matuzahroh N, Fitriani N, Ardiyanti PE, Kuncoro EP, Budiyanto WD, Isnadina DRM, Wahyudianto FE, Radin Mohamed RMS (2020) Behavior of schmutzdecke with varied filtration rates of slow sand filter to remove total coliforms. Heliyon 6:e03736. doi:10.1016/j.heliyon.2020.e03736

Oh S, Hammes F, Liu WT (2018) Metagenomic characterization of biofilter microbial communities in a full-scale drinking water treatment plant. Water Res 128:278–285. doi:10.1016/j.watres.2017.10.054

Pick FC, Fish KE, Biggs CA, Moses JP, Moore G, Boxall JB (2019) Application of enhanced assimilable organic carbon method across operational drinking water systems. PLoS One 14:e0225477. doi:10.1371/journal.pone.0225477

Poghosyan L, Koch H, Frank J, Kessel MAHJ van HJ Van, Cremers G, Alen V, Jetten MSMM, Camp HJMO den MO Den, Lücker S, Hanna Koch, Frank J, Kessel MAHJ van HJ Van, Geert Cremers, Alen T van, Jetten MSMM, Camp HJMO den MO Den, Lücker S (2020) Metagenomic profiling of ammonia- and methane-oxidizing microorganisms in a Dutch drinking water treatment plant. doi:10.1101/2020.05.19.103440Souza

De Souza FH, Roecker PB, Silveira DD, Sens ML, Campos LC (2021) Influence of slow sand filter cleaning process type on filter media biomass : backwashing versus scraping. Water Res

189:116581. doi:10.1016/j.watres.2020.116581

Tatari K, Smets BF, Albrechtsen HJ (2016) Depth investigation of rapid sand filters for drinking water production reveals strong stratification in nitrification biokinetic behavior. Water Res 101:402–410. doi:10.1016/j.watres.2016.04.073

Thiel P, Nolan CP, Scott D, Hiller B, Masters D, Zappia L, Warton B, Nolan P, Alessandrino M, Franzmann P, Heitz A (2006) Activated carbon vs anthracite as primary dual media filters–a pilot plant study. In 69th Annual Water Industry Engineers and Operators' Conference, Bendigo.

Velten S, Boller M, Köster O, Helbing J, Weilenmann HU, Hammes F (2011) Development of biomass in a drinking water granular active carbon (GAC) filter. Water Res 45:6347–6354. doi:10.1016/j.watres.2011.09.017

Wakelin SA, Page DW, Pavelic P, Gregg AL, Dillon PJ (2010) Rich microbial communities inhabit water treatment biofilters and are differentially affected by filter type and sampling depth. Water Sci Technol Water Supply 10:145–156. doi:10.2166/ws.2010.570

Yahya MT, Cluff CB, Gerba CP (1993) Virus removal by slow sand filtration and nanofiltration. Water Sci Technol 27:445–448. doi:10.2166/wst.1993.0389

Yogafanny E, Fuchs S, Obst U (2014) Study of slow sand filtration in removing total coliforms and *E. coli.* J Sains &Teknologi Lingkung 6:107–116. doi:10.20885/jstl.vol6.iss2.art4

Zhang S, Gitungo SW, Axe L, Raczko RF, Dyksen JE (2017) Biologically active filters – an advanced water treatment process for contaminants of emerging concern. Water Res 114:31–41. doi:10.1016/j.watres.2017.02.014
